# Crystal structure, Hirshfeld surface analysis and energy framework study of 6-formyl-7,8,9,11-tetra­hydro-5*H*-pyrido[2,1-*b*]quinazolin-11-one

**DOI:** 10.1107/S2056989020016059

**Published:** 2021-01-01

**Authors:** Akmal Tojiboev, Sherzod Zhurakulov, Valentina Vinogradova, Ulli Englert, Ruimin Wang

**Affiliations:** a Arifov Institute of Ion-Plasma and Laser Technologies of Uzbekistan Academy of Sciences, 100125, Durmon Yuli St. 33, Tashkent, Uzbekistan; b S. Yunusov Institute of Chemistry of Plant Substances, Academy of Sciences of Uzbekistan, Mirzo Ulugbek Str. 77, 100170 Tashkent, Uzbekistan; c National University of Uzbekistan named after Mirzo Ulugbek, 100174, University Str. 4, Olmazor District, Tashkent, Uzbekistan; d Institute of Inorganic Chemistry, RWTH Aachen University, Landoltweg 1, 52056 Aachen, Germany

**Keywords:** Tricyclic quinazoline derivative, intra­molecular N—H⋯O bond, π–π inter­actions, Hirshfeld surface analysis, energy frameworks, crystal structure

## Abstract

At 100 K, the title compound, C_13_H_12_N_2_O_2_, crystallizes in the ortho­rhom­bic space group *Pna*2_1_ with two very similar mol­ecules in the asymmetric unit. An intra­molecular N—H⋯O hydrogen bond leads to an *S*(6) graph-set motif in each of the mol­ecules. Inter­molecular π–π stacking and C=O⋯π inter­actions involving the aldehyde O atoms link mol­ecules into stacks parallel to [100]. A Hirshfeld surface analysis indicates that the most important contributions to the crystal packing stem from H⋯H (49.4%) and H⋯O/O⋯H (21.5%) inter­actions. Energy framework calculations reveal a significant contribution of dispersion energy. The crystal studied was refined as a two-component inversion twin.

## Abstract 2

A short [2.592 (3) Å] intra­molecular N—H⋯O hydrogen bond leads to an *S*(6) graph-set motif. Inter­molecular π–π stacking and C—O⋯π inter­actions dominate the crystal packing.

## Chemical context   

Two major aspects contribute to the inter­est in modified structural analogues of quinazoline alkaloids. On the one hand, they are attractive targets for the development of methods in organic synthesis; reactions sufficiently general to target a wide range of derivatives of a given lead structure should be easy to carry out and warrant high yields. On the other hand, substituted quinazolines allow the study of structure–property relationships with respect to their biological activities (Shakhidoyatov, 1988 ▸; Shakhidoyatov & Elmuradov, 2014 ▸).

The quinazoline alkaloid 7,8,9,11-tetra­hydro-5*H*-pyrido[2,1-*b*]quinazolin-11-one (mackinazolinone alkaloid) was first isolated from the plant Mackinlaya subulata Philipson (Fitzgerald *et al.*, 1966 ▸). A simple method for the synthesis of mackinazolinone *via* condensation of anthranilic acid with δ-valerolactam promoted the use of this compound as a synthon for chemical transformations (Shakhidoyatov *et al.*, 1976 ▸; Oripov *et al.*, 1979 ▸).

**Figure 1:**
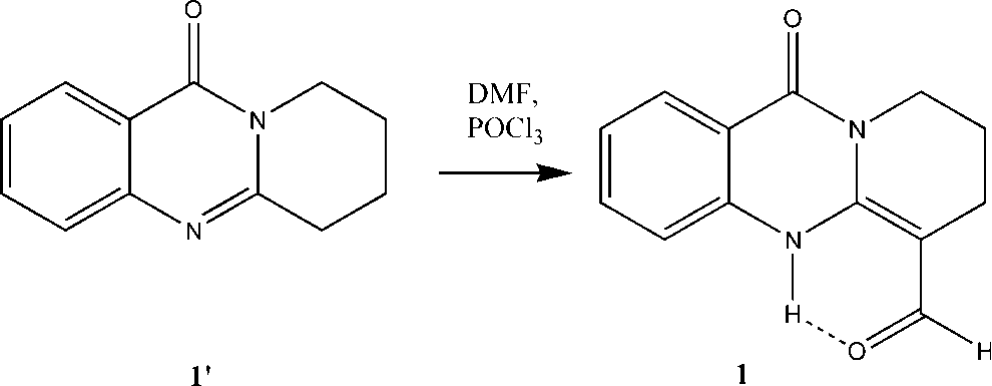
Chemical scheme showing the synthesis of the title compound.

The title compound, 6-formyl-7,8,9,11-tetra­hydro-5*H*-pyrido[2,1-*b*]quinazolin-11-one (**1**) (Fig. 1 ▸), does react with primary amines (Zhurakulov & Vinogradova, 2015 ▸, 2016 ▸), but does not react with pseudoephedrine or 1-(phen­yl)-6,7-dimeth­oxy-1,2,3,4-tetra­hydro­iso­quinoline in a range of solvents with different polarities such as aceto­nitrile, chloro­form, ethanol, tri­fluoro­acetic acid, acetic acid, benzene, DMF or dioxane. The existence of several tautomeric forms for compound (**1**) may be the reason for this selectivity towards primary amines.

Based on ^1^H NMR data and quantum-chemical calculations, Zhurakulov *et al.* (2016 ▸) confirmed that the tautomer with the intra­molecular hydrogen bond represents the energetically favourable form. In order to establish the tautomeric form of (**1**) in the solid state, we studied its mol­ecular and crystal structure. We also report the analysis of the Hirshfeld surface and the energy framework of crystalline (**1**).
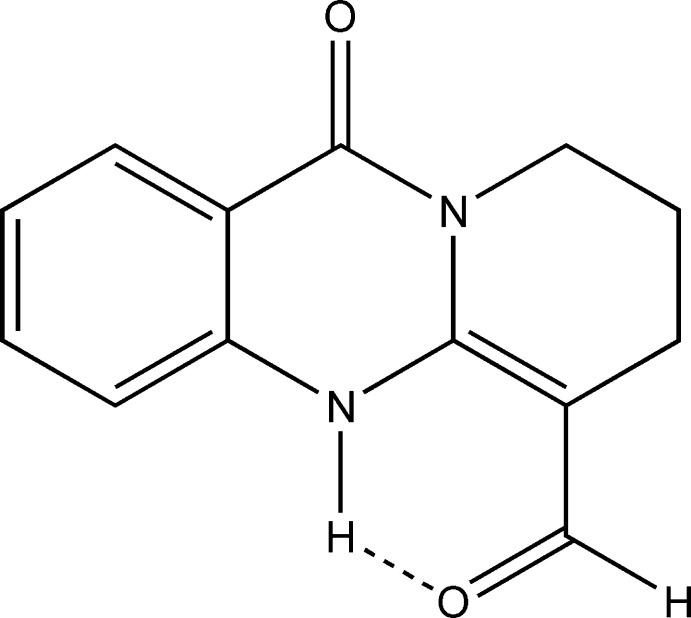


## Structural commentary   

**Figure 2:**
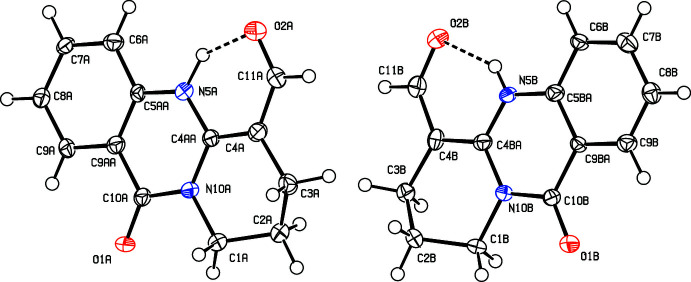
The asymmetric unit of (**1**) with the atom-numbering scheme. Displacement ellipsoids are drawn at the 50% probability level. The intra­molecular N—H⋯O hydrogen bond forming an *S*(6) ring motif is shown with dashed lines.

The asymmetric unit of the title compound contains two mol­ecules *A* and *B* (Fig. 2 ▸). They are almost superimposable, with an r.m.s. of 0.023 Å (Spek, 2020 ▸); an overlay of *A* and *B* is depicted in the supporting information (Fig. S1). In contrast to the quinazolinone moiety, the alkyl ring is not planar. The maximum deviation from the least-squares plane through each of the mol­ecules is encountered for the atoms C2*A* and C2*B* and amounts to 0.515 (3) and 0.521 (3) Å, respectively. The almost coplanar arrangement of the aldehyde group and the pyrimidine ring in either mol­ecule *A* and *B* enables an intra­molecular N—H⋯O inter­action (Table 1 ▸) and formation of an *S*(6) graph-set motif.

**Figure 3:**
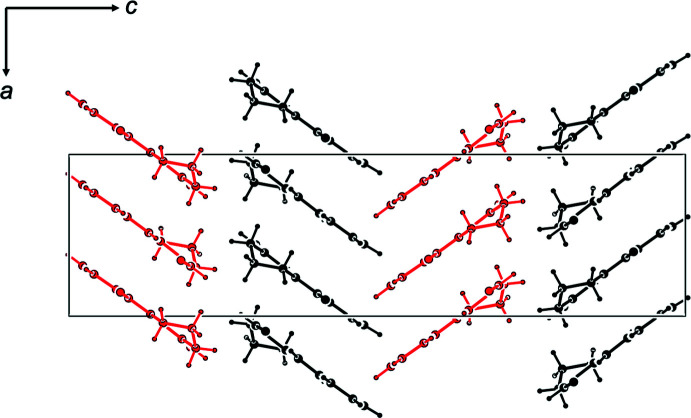
Packing in a view along [010]; the independent mol­ecules *A* (black) and *B* (red) stack into separate columns of equidistant mol­ecules along [100].

Mol­ecules of (**1**) stack into columns parallel to [100] in an equidistant series of coplanar moieties; the independent mol­ecules *A* and *B* segregate into different stacks (Fig. 3 ▸). The intra-stack arrangement does obviously not correspond to translation but involves the *a* glide plane with its mirror component along [010]. The carbonyl groups in subsequent mol­ecules of a stack are therefore oriented alternately in the positive and negative direction of the crystallographic *b* axis, and the same arrangement can be expected for their dipole moments. Although no ‘real’ translation relates consecutive mol­ecules along [100], the rather regular arrangement of essentially planar objects at half a lattice parameter is reflected in moderate pseudosymmetry in reciprocal space: reflection intensities *I_hkl_* are stronger for even indices *h* than for odd ones, with a ratio *I_hkl_, h* = 2*n*: *I_hkl_, h* = (2*n* + 1) of 1.5.

Compound (**1**) crystallizes in the non-centrosymmetric achiral space group *Pna*2_1_, and its absolute structure deserves a comment. The absolute structure is linked to the direction of the polar screw axis along [001]. In the absence of heavy atoms, resonant scattering in (**1**) is minor, with *Friedif* (Flack & Shmueli, 2007 ▸) of 28. We have recently investigated a case of similar low resonant scattering in a Sohnke group, where the absolute structure could be linked to the absolute configuration of the target mol­ecule, and chemical and spectroscopic information could help (Wang & Englert, 2019 ▸). As might be expected, the commonly used indicators for diffraction-based assignment of the absolute structure of (**1**) were associated with rather large standard uncertainties: the Flack parameter (Flack, 1983 ▸) refined to 0.51 (7), and similar results were obtained for Parsons’ quotient method [0.52 (5); Parsons *et al.*, 2013 ▸] and Hooft’s Bayesian analysis [0.51 (5); Hooft *et al.*, 2010 ▸]. All of these indicators suggest that the specimen used for the diffraction experiment was a twin. Refinement converged for a volume ratio of 0.7 (2):0.3 (2) for the twin domains.

## Supra­molecular features   

**Figure 4:**
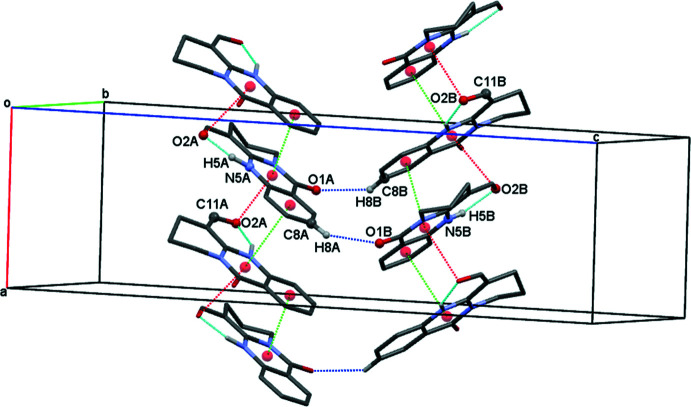
Crystal packing of (**1**) in a view along [100]. Intra­molecular N—H⋯O hydrogen bonds are shown as light-blue and inter­molecular C—H⋯O hydrogen bonds as dark-blue dashed lines. Dashed red lines denote contacts C=O⋯*Cg*1 and C=O⋯*Cg*7 (slippage 1.676 Å for both), and dashed light-green lines *Cg*1⋯*Cg*3 and *Cg*7⋯*Cg*9 contacts. *Cg*3, *Cg*9, *Cg*1 and *Cg*7 correspond to the ring centroids C6*A*–C9*A*/C9*AA*/C5*AA*, C6*B*–C9*B*/C9*BA*/C5*BA*, N5*A*/C4*AA*/N10*A*/C10*A*/C9*AA*/C5*AA* and N5*B*/C4*BA*/N10*B*/C10*B*/C9*BA*/C5*BA*, respectively. For clarity, only H atoms H5*A*, H8*A*, H5*B* and H8*B* are shown.

Consecutive mol­ecules in each column along [100] inter­act *via* π–π stacking and C=O⋯π contacts (Fig. 4 ▸). π–π stacking inter­actions occur between pyrimidine (*Cg*1, *Cg*7) and benzene (*Cg*3, *Cg*9) rings and involve contact distances of *Cg*1⋯*Cg*3(−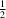 + *x*, 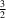 − *y*, *z*) = 3.5154 (18) Å (slippage 0.954 Å) and of *Cg*7⋯*Cg*9(−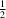 + *x*, 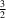 − *y*, *z*) = 3.5159 (19) Å (slippage 1.054 Å).

Mol­ecules within each π-stacked column additionally inter­act *via* C=O⋯π contacts; they amount to C11*A*=O2*A*⋯*Cg*1(*x* + 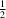, −*y* + 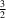, *z*) = 3.212 (2) Å and C11*B*=O2*B*⋯*Cg*7(*x* − 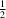, −*y* + 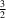, *z*) = 3.215 (2) Å. Perpendicular to the stacking direction, non-classical C—H⋯O hydrogen bonds (Table 1 ▸) link the columns along [001] (Fig. 4 ▸) and thus form layers parallel to (010).

## Hirshfeld surface analysis   

**Figure 5:**
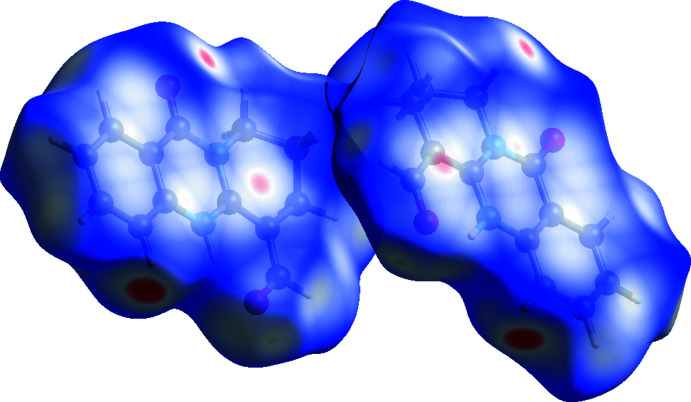
Three-dimensional Hirshfeld surface of the title compound plotted over *d*_norm_ in the range −0.2446 to 1.1709 a.u.

In order to visualize inter­molecular inter­actions in (**1**), the Hirshfeld surface (HS) (Spackman & Jayatilaka, 2009 ▸) was analysed and the associated two-dimensional fingerprint plots (McKinnon *et al.*, 2007 ▸) calculated with *Crystal Explorer 17* (Turner *et al.*, 2017 ▸). The HS mapped with *d*_norm_ is represented in Fig. 5 ▸. White surface areas indicate contacts with distances equal to the sum of van der Waals radii, whereas red and blue colours denote distances shorter (*e.g.* due to hydrogen bonds) or longer than the sum of the van der Waals radii, respectively.

**Figure 6:**
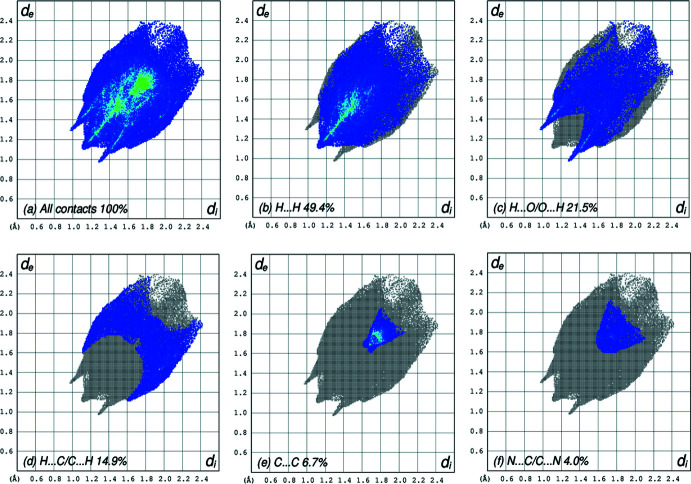
Hirshfeld fingerprint plots for (*a*) all contacts and decomposed into (*b*) H⋯H, (*c*) H⋯O/O⋯H, (*d*) H⋯C/C⋯H, (*e*) C⋯C and (*f*) N⋯C/C⋯N contacts. *d*_i_ and *d*_e_ denote the closest inter­nal and external distances (in Å) from a point on the surface.

The two-dimensional fingerprint plot for all contacts is depicted in Fig. 6 ▸ *a*. H⋯H contacts are responsible for the largest contribution (49.4%) to the Hirshfeld surface (Fig. 6 ▸ *b*). Besides these contacts, H⋯O/O⋯H (21.5%), H⋯C/C⋯H (14.9%), C⋯C (6.7%) and N⋯C/C⋯N (4.0%) inter­actions contribute significantly to the total Hirshfeld surface; their decomposed fingerprint plots are shown in Fig. 6 ▸ *c*–*f*. The contributions of further contacts are only minor and amount to N⋯O/O⋯N (1.4%), C⋯O/O⋯C (1.4%), N⋯H/H⋯N (0.5%) and O⋯O (0.1%).

## Inter­action energy calculations   

**Figure 7:**
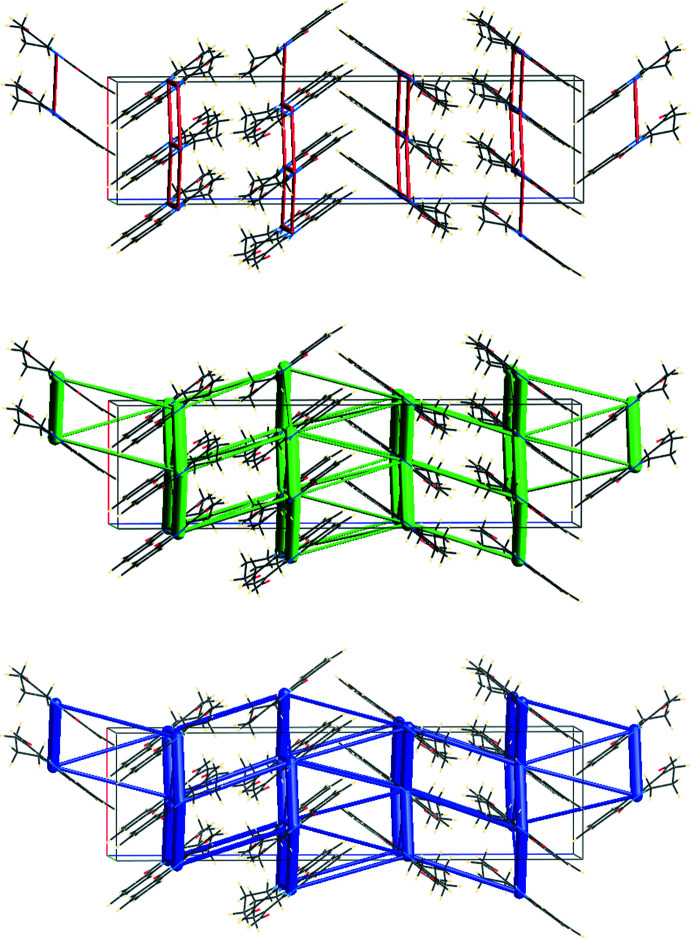
Energy frameworks for the electrostatic (red, top) and dispersion (green, middle) components and the total inter­action energy (blue, bottom). Cylinder radii are proportional to the corresponding energy; a scale factor of 80 and a cut-off value of 10 kJ mol^−1^ have been used.

Inter­molecular inter­action energies were calculated using the CE–HF/3-21G energy model available in *Crystal Explorer 17* (Turner *et al.*, 2017 ▸). The total inter­molecular energy (*E*_tot_) is the sum of electrostatic (*E*_elec_), polarization (*E*_pol_), dispersion (*E*_dis_) and exchange-repulsion (*E*_rep_) energies (Turner *et al.*, 2015 ▸) with scale factors of 1.019, 0.651, 0.901 and 0.811, respectively (Mackenzie *et al.*, 2017 ▸). According to these calculations, the major contribution of −306.5 kJ mol^−1^ is due to dispersion inter­actions (Fig. 7 ▸). The other energy components have values of −91.5 kJ mol^−1^, −37.6 kJ mol^−1^ and 155.7 kJ mol^−1^ for the *E*_elec_, *E*_pol_ and *E*_rep_ energies, respectively. The total inter­action energy resulting from these four components amounts to −267.1 kJ mol^−1^.

## Database survey   

A search in the Cambridge Structural Database (CSD, version 5.41, update January 2020; Groom *et al.*, 2016 ▸) revealed six matches for mol­ecules containing the 3-methyl-2-(propan-2-yl­idene)-2,3-di­hydro­quinazolin-4(1*H*)-one moiety with a similar planar conformation to that in the title structure: 3-(2-methyl­phen­yl)-2-(2-oxo­phenyl­eth­yl)-4(3*H*)-quinazol­inone (FABWUA10; Duke & Codding, 1993 ▸), 3-(2-chloro­phen­yl)-2-[2-oxo-2-(4-pyrid­yl)eth­yl]-4(3*H*)-quinazolinone (FABXAH10; Duke & Codding, 1993 ▸), 2-[2-oxo-2-(4-pyrid­yl)eth­yl]-3-phenyl-4(3*H*)quinazolinone (FABXEL10; Duke & Codding, 1993 ▸), 3-(2-methyl­phen­yl)-2-[2-oxo-2-(4-pyrid­yl)eth­yl]-4(3*H*)-quinazolinone (HADLAZ; Duke & Codding, 1993 ▸), 3-(4-chloro­phen­yl)-2-[2-oxo-2-(4-pyrid­yl)eth­yl]-4(3*H*)-quinazolinone (HADLED; Duke & Codding, 1993 ▸) and (*E*)-2-[2-oxo-2-(thio­phen-2-yl)ethyl­idene]-3-phenyl-2,3-di­hydro­quin­azolin-4(1*H*)-one (SATJOP; Narra *et al.*, 2017 ▸). A search for the 2-amino-1,4,5,6-tetra­hydro­pyridine-3-carbaldehyde moiety gave one hit with similar conformation: 1-methyl-2-(methyl­amino)-1,4,5,6-tetra­hydro­pyridine-3-carbaldehyde (MFHPYM10; Horváth *et al.*, 1983 ▸). Similar to in (**1**), all compounds mentioned above exist as the enamine tautomer in the crystalline state, and their intra­molecular N—H⋯O hydrogen bond between the ethanone and the amine N atom results in an *S*(6) graph set motif.

## Synthesis and crystallization   

Compound (**1**) was synthesized according to the method of Oripov *et al.* (1979 ▸). Yield 12.55 g, 91%; m.p. 474–476 K (after crystallization from hexa­ne), *R*_f_ 0.78 (C_6_H_6_: MeOH 4:1). A detailed report on the synthesis of (**1**) and its characterization by ^1^H NMR is available in Zhurakulov *et al.* (2017 ▸). Crystals suitable for X-ray diffraction were obtained from a methanol solution by slow evaporation of the solvent at room temperature.

## Refinement   

Crystal data, data collection and structure refinement details are summarized in Table 2 ▸. H atoms attached to C were positioned geometrically, with C—H = 0.95 Å (for aromatic), 0.95 Å (for the aldehyde H atom), 0.99 Å (for methyl­ene H atoms) and were refined with *U*_iso_(H) = 1.2*U*_eq_(C). The enamine H atoms H5*A* and H5*B* were refined with a common isotropic displacement parameter; N—H distances were restrained to similarity.

## Supplementary Material

Crystal structure: contains datablock(s) I. DOI: 10.1107/S2056989020016059/wm5590sup1.cif

Structure factors: contains datablock(s) I. DOI: 10.1107/S2056989020016059/wm5590Isup2.hkl

Figure S1. DOI: 10.1107/S2056989020016059/wm5590sup3.tif

Supporting information file. DOI: 10.1107/S2056989020016059/wm5590Isup4.cml

CCDC reference: 2049242

Additional supporting information:  crystallographic information; 3D view; checkCIF report

## Figures and Tables

**Table 1:** Hydrogen-bond geometry (Å, °)

*D*—H⋯*A*	*D*—H	H⋯*A*	*D*⋯*A*	*D*—H⋯*A*
N5*A*—H5*A*⋯O2*A*	0.93 (3)	1.77 (3)	2.592 (3)	146 (3)
N5*B*—H5*B*⋯O2*B*	0.90 (3)	1.82 (3)	2.582 (3)	141 (3)
C1*A*—H1*A*2⋯O1*A* ^i^	0.99	2.57	3.535 (3)	164
C6*A*—H6*A*⋯O1*A* ^ii^	0.95	2.39	3.230 (4)	147
C6*B*—H6*B*⋯O1*B* ^ii^	0.95	2.40	3.239 (4)	148
C8*A*—H8*A*⋯O1*B* ^iii^	0.95	2.60	3.469 (4)	153
C1*B*—H1*B*2⋯O1*B* ^iv^	0.99	2.59	3.550 (3)	164

Symmetry codes: (i) 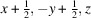; (ii) 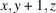; (iii) 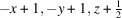; (iv) 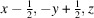.

**Table 2:** Experimental details

Crystal data	
Chemical formula	C_13_H_12_N_2_O_2_
*M*_r_	228.25
Crystal system, space group	Orthorhombic, *P* *n* *a*2_1_
Temperature (K)	100
*a*, *b*, *c* (Å)	8.284 (2), 8.006 (2), 31.637 (6)
*V* (Å^3^)	2098.2 (8)
*Z*	8
Radiation type	Cu *K*α
μ (mm^−1^)	0.81
Crystal size (mm)	0.40 × 0.22 × 0.07

Data collection	
Diffractometer	Stoe Stadivari goniometer, Dectris Pilatus 200K area detector
Absorption correction	Multi-scan (*LANA*; Koziskova *et al.*, 2016 ▸)
*T*_min_, *T*_max_	0.261, 1.000
No. of measured, independent and observed [*I* > 2σ(*I*)] reflections	15842, 3629, 3409
*R*_int_	0.013
(sin θ/λ)_max_ (Å^−1^)	0.619

Refinement	
*R*[*F* ^2^ > 2σ(*F* ^2^)], *wR*(*F* ^2^), *S*	0.036, 0.103, 1.03
No. of reflections	3629
No. of parameters	316
No. of restraints	2
H-atom treatment	H atoms treated by a mixture of independent and constrained refinement
Δρ_max_, Δρ_min_ (e Å^−3^)	0.36, −0.24
Absolute structure	Refined as an inversion twin.
Absolute structure parameter	0.3 (2)

Computer programs: *X-AREA* (Stoe & Cie, 2017 ▸), *SHELXT* (Sheldrick, 2015*a* ▸), *SHELXL2018/3* (Sheldrick, 2015*b* ▸), *PLATON* (Spek, 2020 ▸) and *publCIF* (Westrip, 2010 ▸).

## supplementary crystallographic information

### Crystal data

**Table:**

C_13_H_12_N_2_O_2_	*D*_x_ = 1.445 Mg m^−^^3^
*M**_r_* = 228.25	Cu *K*α radiation, λ = 1.54186 Å
Orthorhombic, *P**n**a*2_1_	Cell parameters from 17372 reflections
*a* = 8.284 (2) Å	θ = 5.6–73.9°
*b* = 8.006 (2) Å	µ = 0.81 mm^−^^1^
*c* = 31.637 (6) Å	*T* = 100 K
*V* = 2098.2 (8) Å^3^	Plate, brown
*Z* = 8	0.40 × 0.22 × 0.07 mm
*F*(000) = 960	

### Data collection

**Table:**

Stoe Stadivari goniometer, Dectris Pilatus 200K area detector diffractometer	3409 reflections with *I* > 2σ(*I*)
Radiation source: XENOCS microsource	*R*_int_ = 0.013
rotation method, ω scans	θ_max_ = 72.8°, θ_min_ = 5.6°
Absorption correction: multi-scan (*LANA*; Koziskova *et al.*, 2016)	*h* = −7→10
*T*_min_ = 0.261, *T*_max_ = 1.000	*k* = −7→9
15842 measured reflections	*l* = −35→38
3629 independent reflections	

### Refinement

**Table:**

Refinement on *F*^2^	H atoms treated by a mixture of independent and constrained refinement
Least-squares matrix: full	*w* = 1/[σ^2^(*F*_o_^2^) + (0.0861*P*)^2^ + 0.1201*P*] where *P* = (*F*_o_^2^ + 2*F*_c_^2^)/3
*R*[*F*^2^ > 2σ(*F*^2^)] = 0.036	(Δ/σ)_max_ = 0.004
*wR*(*F*^2^) = 0.103	Δρ_max_ = 0.36 e Å^−^^3^
*S* = 1.03	Δρ_min_ = −0.24 e Å^−^^3^
3629 reflections	Extinction correction: SHELXL-2018/3 (Sheldrick, 2015b), Fc^*^=kFc[1+0.001xFc^2^λ^3^/sin(2θ)]^-1/4^
316 parameters	Extinction coefficient: 0.0008 (2)
2 restraints	Absolute structure: Refined as an inversion twin.
Primary atom site location: dual	Absolute structure parameter: 0.3 (2)
Hydrogen site location: mixed	

### Special details

**Table:**

Geometry. All esds (except the esd in the dihedral angle between two l.s. planes) are estimated using the full covariance matrix. The cell esds are taken into account individually in the estimation of esds in distances, angles and torsion angles; correlations between esds in cell parameters are only used when they are defined by crystal symmetry. An approximate (isotropic) treatment of cell esds is used for estimating esds involving l.s. planes.
Refinement. Refined as a two-component inversion twin

### Fractional atomic coordinates and isotropic or equivalent isotropic displacement parameters (Å^2^)

**Table:**

	*x*	*y*	*z*	*U*_iso_*/*U*_eq_	
O1A	0.6021 (2)	0.3190 (2)	0.91582 (6)	0.0238 (4)	
O2A	0.9070 (2)	0.9397 (2)	0.81869 (6)	0.0273 (4)	
N10A	0.7388 (2)	0.4746 (2)	0.86753 (6)	0.0188 (4)	
N5A	0.7374 (4)	0.7667 (3)	0.87182 (9)	0.0205 (6)	
H5A	0.785 (4)	0.861 (3)	0.8601 (12)	0.031 (5)*	
C10A	0.6394 (3)	0.4572 (3)	0.90289 (7)	0.0193 (4)	
C4AA	0.7900 (3)	0.6267 (3)	0.85180 (7)	0.0183 (5)	
C5AA	0.6354 (3)	0.7651 (3)	0.90621 (10)	0.0156 (6)	
C9AA	0.5867 (3)	0.6135 (3)	0.92277 (9)	0.0199 (6)	
C9A	0.4876 (4)	0.6078 (3)	0.95915 (9)	0.0198 (6)	
H9A	0.450928	0.504197	0.970188	0.024*	
C8A	0.4454 (4)	0.7578 (3)	0.97834 (12)	0.0208 (7)	
H8A	0.386242	0.756350	1.004070	0.025*	
C7A	0.4889 (4)	0.9111 (4)	0.96020 (9)	0.0201 (5)	
H7A	0.450755	1.012023	0.972419	0.024*	
C6A	0.5870 (3)	0.9174 (4)	0.92456 (9)	0.0221 (5)	
H6A	0.620410	1.021297	0.912959	0.027*	
C4A	0.8927 (3)	0.6410 (3)	0.81695 (8)	0.0219 (5)	
C11A	0.9422 (3)	0.7999 (4)	0.80294 (8)	0.0236 (5)	
H11A	1.010208	0.801898	0.778758	0.028*	
C3A	0.9495 (3)	0.4844 (3)	0.79423 (7)	0.0251 (5)	
H3A1	1.055458	0.449174	0.805650	0.030*	
H3A2	0.962786	0.508158	0.763718	0.030*	
C2A	0.8267 (3)	0.3449 (3)	0.80027 (8)	0.0261 (5)	
H2A1	0.725049	0.374290	0.785613	0.031*	
H2A2	0.868683	0.240345	0.787679	0.031*	
C1A	0.7936 (3)	0.3181 (3)	0.84685 (7)	0.0234 (5)	
H1A1	0.709624	0.231135	0.850210	0.028*	
H1A2	0.893143	0.277918	0.860881	0.028*	
O1B	0.6478 (2)	0.3186 (2)	0.58347 (6)	0.0246 (4)	
O2B	0.3447 (2)	0.9370 (2)	0.68172 (6)	0.0260 (4)	
N10B	0.5127 (2)	0.4739 (2)	0.63232 (6)	0.0182 (4)	
N5B	0.5164 (4)	0.7649 (2)	0.62934 (9)	0.0175 (5)	
H5B	0.482 (4)	0.861 (3)	0.6411 (12)	0.031 (5)*	
C10B	0.6112 (3)	0.4574 (3)	0.59674 (7)	0.0188 (4)	
C4BA	0.4630 (3)	0.6258 (3)	0.64828 (8)	0.0186 (5)	
C5BA	0.6149 (4)	0.7667 (3)	0.59430 (11)	0.0206 (7)	
C9BA	0.6647 (3)	0.6125 (3)	0.57672 (8)	0.0166 (5)	
C9B	0.7593 (4)	0.6123 (3)	0.54056 (10)	0.0214 (6)	
H9B	0.788639	0.508758	0.528072	0.026*	
C8B	0.8117 (4)	0.7591 (3)	0.52233 (13)	0.0232 (8)	
H8B	0.880004	0.757998	0.498217	0.028*	
C7B	0.7611 (4)	0.9097 (4)	0.54040 (10)	0.0244 (6)	
H7B	0.793649	1.011674	0.527607	0.029*	
C6B	0.6666 (3)	0.9156 (4)	0.57585 (9)	0.0196 (5)	
H6B	0.636423	1.020004	0.587748	0.023*	
C4B	0.3593 (3)	0.6378 (3)	0.68323 (8)	0.0205 (5)	
C11B	0.3090 (3)	0.7967 (4)	0.69727 (8)	0.0234 (4)	
H11B	0.239986	0.798223	0.721255	0.028*	
C3B	0.3026 (3)	0.4828 (3)	0.70566 (7)	0.0238 (5)	
H3B1	0.196540	0.447861	0.694240	0.029*	
H3B2	0.289541	0.506169	0.736197	0.029*	
C2B	0.4255 (3)	0.3433 (3)	0.69938 (8)	0.0254 (5)	
H2B1	0.527309	0.372514	0.714023	0.030*	
H2B2	0.383741	0.238507	0.711861	0.030*	
C1B	0.4583 (3)	0.3175 (3)	0.65270 (7)	0.0226 (5)	
H1B1	0.542250	0.230628	0.649173	0.027*	
H1B2	0.358647	0.277673	0.638669	0.027*	

### Atomic displacement parameters (Å^2^)

**Table:**

	*U*^11^	*U*^22^	*U*^33^	*U*^12^	*U*^13^	*U*^23^
O1A	0.0349 (8)	0.0136 (9)	0.0228 (8)	−0.0017 (6)	0.0023 (6)	0.0010 (5)
O2A	0.0321 (9)	0.0214 (9)	0.0282 (10)	−0.0033 (7)	−0.0001 (7)	−0.0003 (8)
N10A	0.0238 (8)	0.0142 (9)	0.0185 (9)	0.0011 (7)	−0.0015 (7)	−0.0010 (7)
N5A	0.0249 (14)	0.0170 (11)	0.0196 (13)	−0.0012 (7)	−0.0001 (11)	−0.0019 (7)
C10A	0.0225 (10)	0.0184 (11)	0.0170 (10)	0.0010 (7)	−0.0047 (8)	−0.0013 (7)
C4AA	0.0207 (10)	0.0172 (12)	0.0171 (11)	0.0013 (8)	−0.0046 (8)	0.0005 (8)
C5AA	0.0155 (14)	0.0179 (12)	0.0133 (12)	0.0008 (7)	−0.0040 (10)	0.0000 (8)
C9AA	0.0217 (12)	0.0192 (15)	0.0189 (12)	0.0017 (8)	−0.0050 (11)	−0.0019 (10)
C9A	0.0225 (12)	0.0216 (14)	0.0153 (11)	0.0016 (9)	−0.0026 (9)	0.0015 (9)
C8A	0.0249 (17)	0.0211 (16)	0.0165 (17)	0.0007 (8)	−0.0021 (11)	−0.0021 (7)
C7A	0.0251 (12)	0.0166 (12)	0.0185 (12)	0.0035 (10)	−0.0036 (10)	−0.0042 (10)
C6A	0.0246 (12)	0.0179 (13)	0.0237 (12)	0.0008 (10)	−0.0052 (11)	0.0000 (10)
C4A	0.0239 (12)	0.0204 (12)	0.0214 (12)	0.0025 (10)	−0.0023 (9)	−0.0030 (10)
C11A	0.0248 (11)	0.0254 (13)	0.0205 (12)	−0.0006 (11)	0.0001 (9)	0.0032 (11)
C3A	0.0299 (11)	0.0247 (12)	0.0208 (12)	0.0057 (9)	0.0040 (8)	0.0022 (9)
C2A	0.0370 (12)	0.0216 (10)	0.0197 (12)	0.0022 (9)	0.0011 (8)	−0.0028 (8)
C1A	0.0317 (11)	0.0180 (13)	0.0204 (13)	0.0027 (9)	0.0008 (9)	−0.0022 (8)
O1B	0.0348 (8)	0.0165 (9)	0.0226 (8)	0.0016 (6)	0.0030 (6)	−0.0001 (5)
O2B	0.0311 (8)	0.0213 (8)	0.0256 (9)	0.0023 (8)	0.0014 (6)	−0.0046 (7)
N10B	0.0237 (9)	0.0141 (9)	0.0169 (8)	−0.0007 (7)	−0.0017 (7)	0.0009 (7)
N5B	0.0209 (12)	0.0146 (11)	0.0170 (12)	0.0002 (7)	−0.0024 (10)	−0.0021 (7)
C10B	0.0228 (10)	0.0159 (10)	0.0176 (10)	0.0004 (8)	−0.0030 (8)	−0.0006 (7)
C4BA	0.0213 (10)	0.0150 (12)	0.0194 (11)	0.0006 (7)	−0.0065 (8)	−0.0005 (8)
C5BA	0.0243 (17)	0.0169 (13)	0.0205 (15)	0.0008 (8)	−0.0056 (12)	−0.0005 (9)
C9BA	0.0197 (11)	0.0139 (14)	0.0163 (11)	−0.0004 (8)	−0.0030 (10)	0.0017 (8)
C9B	0.0228 (12)	0.0178 (13)	0.0235 (13)	0.0027 (9)	−0.0029 (10)	−0.0008 (10)
C8B	0.0207 (16)	0.0280 (17)	0.0209 (19)	−0.0026 (8)	−0.0001 (11)	0.0022 (8)
C7B	0.0235 (13)	0.0231 (13)	0.0265 (14)	−0.0021 (11)	−0.0043 (11)	0.0065 (11)
C6B	0.0238 (12)	0.0144 (12)	0.0205 (12)	0.0001 (9)	−0.0038 (10)	0.0025 (9)
C4B	0.0200 (11)	0.0262 (13)	0.0154 (10)	−0.0002 (9)	−0.0031 (8)	−0.0023 (9)
C11B	0.0240 (11)	0.0261 (12)	0.0201 (12)	0.0003 (10)	−0.0019 (8)	−0.0017 (11)
C3B	0.0314 (11)	0.0215 (11)	0.0185 (11)	−0.0044 (9)	0.0031 (8)	0.0004 (8)
C2B	0.0380 (12)	0.0210 (10)	0.0170 (11)	−0.0012 (9)	0.0015 (9)	0.0035 (7)
C1B	0.0330 (11)	0.0129 (12)	0.0220 (12)	−0.0020 (8)	0.0005 (9)	0.0021 (8)

### Geometric parameters (Å, º)

**Table:**

O1A—C10A	1.220 (3)	O1B—C10B	1.226 (3)
O2A—C11A	1.259 (4)	O2B—C11B	1.262 (4)
N10A—C4AA	1.382 (3)	N10B—C4BA	1.380 (3)
N10A—C10A	1.396 (3)	N10B—C10B	1.397 (3)
N10A—C1A	1.485 (3)	N10B—C1B	1.478 (3)
N5A—C4AA	1.359 (3)	N5B—C4BA	1.340 (3)
N5A—C5AA	1.377 (4)	N5B—C5BA	1.377 (5)
N5A—H5A	0.93 (2)	N5B—H5B	0.90 (2)
C10A—C9AA	1.467 (3)	C10B—C9BA	1.463 (3)
C4AA—C4A	1.397 (4)	C4BA—C4B	1.404 (4)
C5AA—C9AA	1.382 (4)	C5BA—C6B	1.395 (4)
C5AA—C6A	1.409 (4)	C5BA—C9BA	1.415 (4)
C9AA—C9A	1.414 (4)	C9BA—C9B	1.386 (4)
C9A—C8A	1.390 (4)	C9B—C8B	1.380 (4)
C9A—H9A	0.9500	C9B—H9B	0.9500
C8A—C7A	1.402 (4)	C8B—C7B	1.398 (4)
C8A—H8A	0.9500	C8B—H8B	0.9500
C7A—C6A	1.391 (4)	C7B—C6B	1.369 (4)
C7A—H7A	0.9500	C7B—H7B	0.9500
C6A—H6A	0.9500	C6B—H6B	0.9500
C4A—C11A	1.409 (4)	C4B—C11B	1.410 (4)
C4A—C3A	1.520 (3)	C4B—C3B	1.505 (3)
C11A—H11A	0.9500	C11B—H11B	0.9500
C3A—C2A	1.523 (3)	C3B—C2B	1.524 (3)
C3A—H3A1	0.9900	C3B—H3B1	0.9900
C3A—H3A2	0.9900	C3B—H3B2	0.9900
C2A—C1A	1.514 (3)	C2B—C1B	1.516 (3)
C2A—H2A1	0.9900	C2B—H2B1	0.9900
C2A—H2A2	0.9900	C2B—H2B2	0.9900
C1A—H1A1	0.9900	C1B—H1B1	0.9900
C1A—H1A2	0.9900	C1B—H1B2	0.9900
			
C4AA—N10A—C10A	123.88 (18)	C4BA—N10B—C10B	123.5 (2)
C4AA—N10A—C1A	119.42 (18)	C4BA—N10B—C1B	119.72 (19)
C10A—N10A—C1A	116.69 (17)	C10B—N10B—C1B	116.73 (18)
C4AA—N5A—C5AA	123.9 (2)	C4BA—N5B—C5BA	124.3 (2)
C4AA—N5A—H5A	110 (2)	C4BA—N5B—H5B	115 (3)
C5AA—N5A—H5A	126 (2)	C5BA—N5B—H5B	121 (3)
O1A—C10A—N10A	120.6 (2)	O1B—C10B—N10B	120.4 (2)
O1A—C10A—C9AA	123.7 (2)	O1B—C10B—C9BA	123.1 (2)
N10A—C10A—C9AA	115.7 (2)	N10B—C10B—C9BA	116.5 (2)
N5A—C4AA—N10A	117.4 (2)	N5B—C4BA—N10B	118.1 (2)
N5A—C4AA—C4A	119.7 (2)	N5B—C4BA—C4B	119.8 (2)
N10A—C4AA—C4A	122.9 (2)	N10B—C4BA—C4B	122.1 (2)
N5A—C5AA—C9AA	119.1 (2)	N5B—C5BA—C6B	121.8 (3)
N5A—C5AA—C6A	119.5 (2)	N5B—C5BA—C9BA	118.7 (2)
C9AA—C5AA—C6A	121.4 (3)	C6B—C5BA—C9BA	119.4 (3)
C5AA—C9AA—C9A	120.4 (2)	C9B—C9BA—C5BA	119.3 (2)
C5AA—C9AA—C10A	120.0 (2)	C9B—C9BA—C10B	121.8 (2)
C9A—C9AA—C10A	119.6 (2)	C5BA—C9BA—C10B	118.8 (2)
C8A—C9A—C9AA	118.3 (3)	C8B—C9B—C9BA	121.5 (3)
C8A—C9A—H9A	120.9	C8B—C9B—H9B	119.3
C9AA—C9A—H9A	120.9	C9BA—C9B—H9B	119.3
C9A—C8A—C7A	120.8 (3)	C9B—C8B—C7B	118.0 (4)
C9A—C8A—H8A	119.6	C9B—C8B—H8B	121.0
C7A—C8A—H8A	119.6	C7B—C8B—H8B	121.0
C6A—C7A—C8A	120.9 (3)	C6B—C7B—C8B	122.4 (3)
C6A—C7A—H7A	119.5	C6B—C7B—H7B	118.8
C8A—C7A—H7A	119.5	C8B—C7B—H7B	118.8
C7A—C6A—C5AA	117.9 (3)	C7B—C6B—C5BA	119.3 (3)
C7A—C6A—H6A	121.0	C7B—C6B—H6B	120.4
C5AA—C6A—H6A	121.0	C5BA—C6B—H6B	120.4
C4AA—C4A—C11A	120.0 (2)	C4BA—C4B—C11B	119.4 (2)
C4AA—C4A—C3A	119.6 (2)	C4BA—C4B—C3B	120.4 (2)
C11A—C4A—C3A	120.4 (3)	C11B—C4B—C3B	120.2 (2)
O2A—C11A—C4A	127.6 (2)	O2B—C11B—C4B	127.6 (2)
O2A—C11A—H11A	116.2	O2B—C11B—H11B	116.2
C4A—C11A—H11A	116.2	C4B—C11B—H11B	116.2
C4A—C3A—C2A	109.80 (19)	C4B—C3B—C2B	109.50 (19)
C4A—C3A—H3A1	109.7	C4B—C3B—H3B1	109.8
C2A—C3A—H3A1	109.7	C2B—C3B—H3B1	109.8
C4A—C3A—H3A2	109.7	C4B—C3B—H3B2	109.8
C2A—C3A—H3A2	109.7	C2B—C3B—H3B2	109.8
H3A1—C3A—H3A2	108.2	H3B1—C3B—H3B2	108.2
C1A—C2A—C3A	110.31 (19)	C1B—C2B—C3B	110.27 (19)
C1A—C2A—H2A1	109.6	C1B—C2B—H2B1	109.6
C3A—C2A—H2A1	109.6	C3B—C2B—H2B1	109.6
C1A—C2A—H2A2	109.6	C1B—C2B—H2B2	109.6
C3A—C2A—H2A2	109.6	C3B—C2B—H2B2	109.6
H2A1—C2A—H2A2	108.1	H2B1—C2B—H2B2	108.1
N10A—C1A—C2A	111.38 (18)	N10B—C1B—C2B	111.35 (18)
N10A—C1A—H1A1	109.4	N10B—C1B—H1B1	109.4
C2A—C1A—H1A1	109.4	C2B—C1B—H1B1	109.4
N10A—C1A—H1A2	109.4	N10B—C1B—H1B2	109.4
C2A—C1A—H1A2	109.4	C2B—C1B—H1B2	109.4
H1A1—C1A—H1A2	108.0	H1B1—C1B—H1B2	108.0
			
C4AA—N10A—C10A—O1A	−178.06 (19)	C4BA—N10B—C10B—O1B	−178.11 (19)
C1A—N10A—C10A—O1A	0.6 (3)	C1B—N10B—C10B—O1B	0.6 (3)
C4AA—N10A—C10A—C9AA	1.4 (3)	C4BA—N10B—C10B—C9BA	1.3 (3)
C1A—N10A—C10A—C9AA	−179.96 (19)	C1B—N10B—C10B—C9BA	−179.99 (19)
C5AA—N5A—C4AA—N10A	−0.8 (4)	C5BA—N5B—C4BA—N10B	1.5 (4)
C5AA—N5A—C4AA—C4A	179.6 (3)	C5BA—N5B—C4BA—C4B	−178.3 (3)
C10A—N10A—C4AA—N5A	−1.1 (3)	C10B—N10B—C4BA—N5B	−2.1 (3)
C1A—N10A—C4AA—N5A	−179.7 (2)	C1B—N10B—C4BA—N5B	179.2 (2)
C10A—N10A—C4AA—C4A	178.4 (2)	C10B—N10B—C4BA—C4B	177.7 (2)
C1A—N10A—C4AA—C4A	−0.2 (3)	C1B—N10B—C4BA—C4B	−1.0 (3)
C4AA—N5A—C5AA—C9AA	2.4 (4)	C4BA—N5B—C5BA—C6B	179.9 (3)
C4AA—N5A—C5AA—C6A	−179.6 (3)	C4BA—N5B—C5BA—C9BA	−0.1 (5)
N5A—C5AA—C9AA—C9A	177.3 (3)	N5B—C5BA—C9BA—C9B	177.7 (3)
C6A—C5AA—C9AA—C9A	−0.7 (5)	C6B—C5BA—C9BA—C9B	−2.3 (5)
N5A—C5AA—C9AA—C10A	−2.0 (4)	N5B—C5BA—C9BA—C10B	−0.7 (4)
C6A—C5AA—C9AA—C10A	−179.9 (2)	C6B—C5BA—C9BA—C10B	179.2 (2)
O1A—C10A—C9AA—C5AA	179.6 (2)	O1B—C10B—C9BA—C9B	1.1 (4)
N10A—C10A—C9AA—C5AA	0.2 (3)	N10B—C10B—C9BA—C9B	−178.3 (2)
O1A—C10A—C9AA—C9A	0.4 (4)	O1B—C10B—C9BA—C5BA	179.5 (2)
N10A—C10A—C9AA—C9A	−179.1 (2)	N10B—C10B—C9BA—C5BA	0.2 (3)
C5AA—C9AA—C9A—C8A	−2.0 (4)	C5BA—C9BA—C9B—C8B	2.8 (5)
C10A—C9AA—C9A—C8A	177.3 (3)	C10B—C9BA—C9B—C8B	−178.8 (3)
C9AA—C9A—C8A—C7A	5.1 (5)	C9BA—C9B—C8B—C7B	−2.6 (6)
C9A—C8A—C7A—C6A	−5.5 (5)	C9B—C8B—C7B—C6B	1.9 (6)
C8A—C7A—C6A—C5AA	2.7 (4)	C8B—C7B—C6B—C5BA	−1.5 (5)
N5A—C5AA—C6A—C7A	−177.7 (3)	N5B—C5BA—C6B—C7B	−178.4 (3)
C9AA—C5AA—C6A—C7A	0.3 (5)	C9BA—C5BA—C6B—C7B	1.7 (5)
N5A—C4AA—C4A—C11A	0.5 (4)	N5B—C4BA—C4B—C11B	1.7 (4)
N10A—C4AA—C4A—C11A	−179.0 (2)	N10B—C4BA—C4B—C11B	−178.1 (2)
N5A—C4AA—C4A—C3A	−179.2 (2)	N5B—C4BA—C4B—C3B	−178.1 (2)
N10A—C4AA—C4A—C3A	1.3 (3)	N10B—C4BA—C4B—C3B	2.1 (3)
C4AA—C4A—C11A—O2A	1.3 (4)	C4BA—C4B—C11B—O2B	0.8 (4)
C3A—C4A—C11A—O2A	−179.1 (2)	C3B—C4B—C11B—O2B	−179.4 (2)
C4AA—C4A—C3A—C2A	26.3 (3)	C4BA—C4B—C3B—C2B	26.0 (3)
C11A—C4A—C3A—C2A	−153.4 (2)	C11B—C4B—C3B—C2B	−153.9 (2)
C4A—C3A—C2A—C1A	−54.1 (3)	C4B—C3B—C2B—C1B	−54.1 (3)
C4AA—N10A—C1A—C2A	−28.9 (3)	C4BA—N10B—C1B—C2B	−28.5 (3)
C10A—N10A—C1A—C2A	152.38 (19)	C10B—N10B—C1B—C2B	152.68 (19)
C3A—C2A—C1A—N10A	56.2 (2)	C3B—C2B—C1B—N10B	56.2 (2)

### Hydrogen-bond geometry (Å, º)

**Table:**

*D*—H···*A*	*D*—H	H···*A*	*D*···*A*	*D*—H···*A*
N5*A*—H5*A*···O2*A*	0.93 (3)	1.77 (3)	2.592 (3)	146 (3)
N5*B*—H5*B*···O2*B*	0.90 (3)	1.82 (3)	2.582 (3)	141 (3)
C1*A*—H1*A*2···O1*A*^i^	0.99	2.57	3.535 (3)	164
C6*A*—H6*A*···O1*A*^ii^	0.95	2.39	3.230 (4)	147
C6*B*—H6*B*···O1*B*^ii^	0.95	2.40	3.239 (4)	148
C8*A*—H8*A*···O1*B*^iii^	0.95	2.60	3.469 (4)	153
C1*B*—H1*B*2···O1*B*^iv^	0.99	2.59	3.550 (3)	164

Symmetry codes: (i) *x*+1/2, −*y*+1/2, *z*; (ii) *x*, *y*+1, *z*; (iii) −*x*+1, −*y*+1, *z*+1/2; (iv) *x*−1/2, −*y*+1/2, *z*.
