# Supplementary figures and images for: Crystal structure, Hirshfeld surface analysis and energy framework study of 6-formyl-7,8,9,11-tetra­hydro-5H-pyrido[2,1-b]quinazolin-11-one
Source: Acta Crystallogr E Crystallogr Commun. 2021 Jan 1;77(Pt 1):47–51. doi: 10.1107/S2056989020016059 (PMC7784053; doi:10.1107/S2056989020016059)

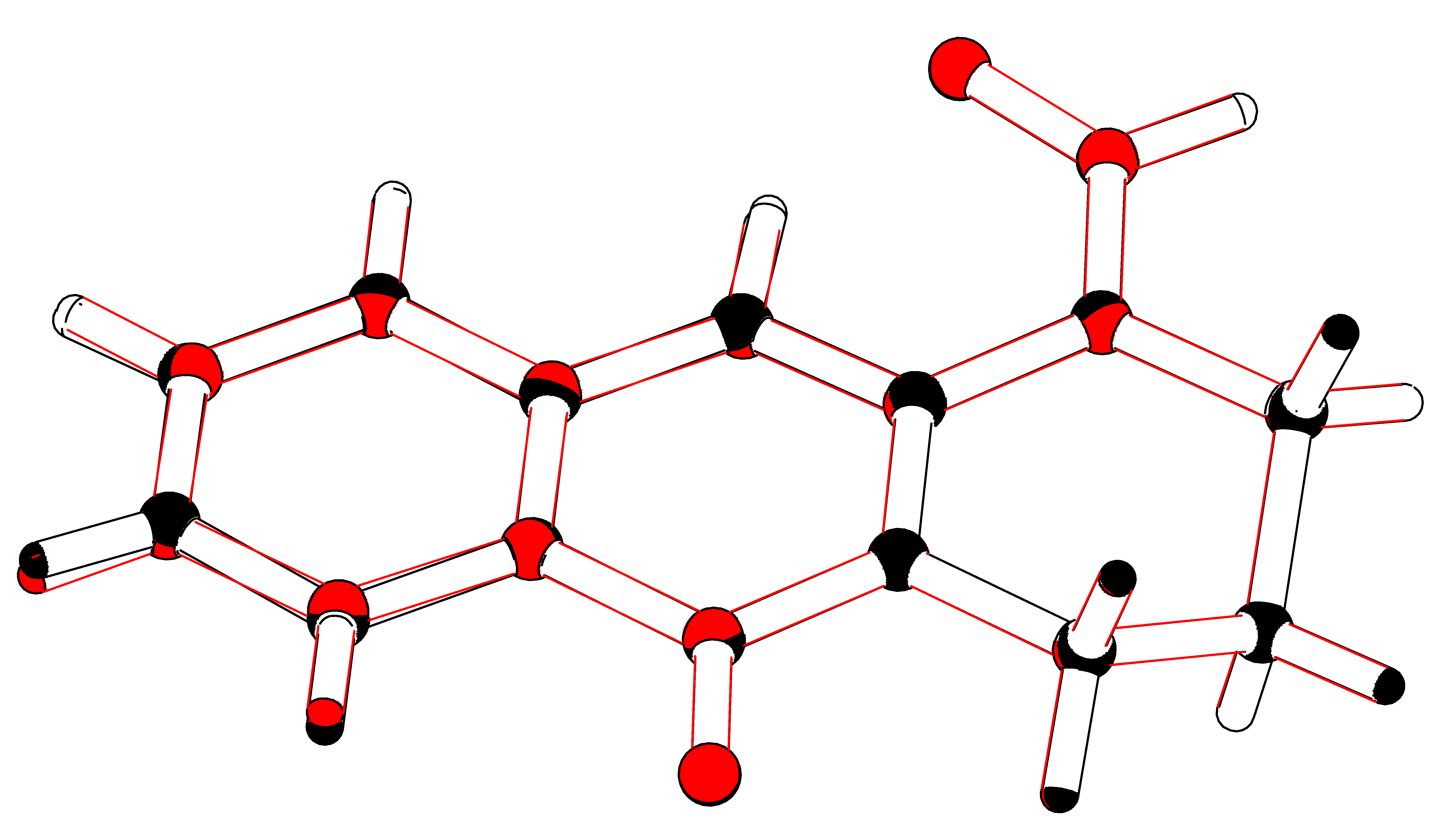

Supplement: Supplementary file 3 [file e-77-00047-sup3.tif]
